# Preoperative radiomics model using gadobenate dimeglumine-enhanced magnetic resonance imaging for predicting β-catenin mutation in patients with hepatocellular carcinoma: A retrospective study

**DOI:** 10.3389/fonc.2022.916126

**Published:** 2022-09-16

**Authors:** Fengxia Zeng, Hui Dai, Xu Li, Le Guo, Ningyang Jia, Jun Yang, Danping Huang, Hui Zeng, Weiguo Chen, Ling Zhang, Genggeng Qin

**Affiliations:** ^1^Department of Radiology, Nanfang Hospital, Southern Medical University, Guangzhou, China; ^2^Hospital Office, Ganzhou People’s Hospital, Ganzhou, China; ^3^Hospital Office, Ganzhou Hospital-Nanfang Hospital, Southern Medical University, Ganzhou, China; ^4^School of Biomedical Engineering, Southern Medical University, Guangzhou, China; ^5^Department of Radiology, Eastern Hepatobiliary Surgery Hospital, Second Military Medical University, Shanghai, China; ^6^Department of Radiology, Ganzhou Hospital-Nanfang Hospital, Southern Medical University, Ganzhou, China

**Keywords:** hepatocellular carcinoma, β-catenin mutation, magnetic resonance imaging, Gd-BOPTA, radiomics

## Abstract

**Objective:**

To compare and evaluate radiomics models to preoperatively predict β-catenin mutation in patients with hepatocellular carcinoma (HCC).

**Methods:**

Ninety-eight patients who underwent preoperative gadobenate dimeglumine (Gd-BOPTA)-enhanced MRI were retrospectively included. Volumes of interest were manually delineated on arterial phase, portal venous phase, delay phase, and hepatobiliary phase (HBP) images. Radiomics features extracted from different combinations of imaging phases were analyzed and validated. A linear support vector classifier was applied to develop different models.

**Results:**

Among all 15 types of radiomics models, the model with the best performance was seen in the R^HBP^ radiomics model. The area under the receiver operating characteristic curve (AUC), accuracy, sensitivity, specificity of the R^HBP^ radiomics model in the training and validation cohorts were 0.86 (95% confidence interval [CI], 0.75–0.93), 0.75, 1.0, and 0.65 and 0.82 (95% CI, 0.63–0.93), 0.73, 0.67, and 0.76, respectively. The combined model integrated radiomics features in the R^HBP^ radiomics model, and signatures in the clinical model did not improve further compared to the single HBP radiomics model with AUCs of 0.86 and 0.76. Good calibration for the best R^HBP^ radiomics model was displayed in both cohorts; the decision curve showed that the net benefit could achieve 0.15. The most important radiomics features were low and high gray-level zone emphases based on gray-level size zone matrix with the same Shapley additive explanation values of 0.424.

**Conclusion:**

The R^HBP^ radiomics model may be used as an effective model indicative of HCCs with β-catenin mutation preoperatively and thus could guide personalized medicine.

## Highlights

1. β-catenin can be preoperatively estimated using radiomics model based on Gd-BOPTA-enhanced MRI.
2. Among all 15 types of radiomics models, the model with the best performance was seen in the R^HBP^ radiomics model.
3. The R^HBP^ radiomics model may assist in the selection of appropriate decision-making for personalized medicine in patients with hepatocellular carcinoma.

## Introduction

Hepatocellular carcinoma (HCC) has become the third most common cause of cancer-related deaths globally in 2020, making it a health problem worldwide (1). Although there has been recent progress in the treatment of HCC, the 5-year tumor recurrence occurs in approximately 35% of cases after liver transplantation, and 70% after hepatectomy indicates an unsatisfactory overall survival for patients with HCC (2–4). Additionally, patients with advanced HCC are not eligible for curative therapies. Thus, immunotherapy plays a critical role in HCC (5, 6).

Recently, programmed cell death 1 (PD-1) immune checkpoint inhibitors have shown efficacy in patients with HCC at advanced stages (7, 8). Anti-PD-1 therapy has revealed unprecedented response and disease control rates in clinical trials and has become the second-line therapy for HCC treatment granted by the Food and Drug Administration (9–11). Unfortunately, some patients failed to respond effectively, and not all patients showed positive results on prognosis, although excellent antitumor responses were observed with anti-PD-1 therapy (12, 13). Hence, selecting the subgroup that would receive the best benefit from anti-PD-1 antibody is important in the management of patients with HCC (13).

β-catenin activation can induce immune escape and resistance to anti-PD-1 therapy in HCC cases (14–17). Morita et al. (16) also showed that β-catenin without mutation was significantly correlated with longer survival in both progression-free survival and overall survival with anti-PD-1 therapy. The β-catenin mutation, which contributes to the activation of the Wnt/β-catenin signaling pathway, can be observed in approximately 30%–40% of patients with HCC (18). β-catenin, which is an intracellular signal transducer in the WNT signaling pathway, is encoded by CTNNB1 and closely related to the occurrence and development of liver tumors. Most liver tumors have mutations in genes encoding key components of the WNT/β-catenin signaling pathway (14, 18, 19). Studies have reported that the Wnt/β-catenin pathway is correlated with carcinogenesis, especially in hepatocellular adenoma (20, 21). However, in patients with HCC, presence of the β-catenin mutation may suggest better cell differentiation and a more favorable prognosis (22, 23). Currently, the diagnosis of β-catenin mutation depends on polymerase chain reaction or immunohistochemical analysis. Nuclear expression of β-catenin in immunohistochemical analysis can hint at the β-catenin mutation and activation of the β-catenin pathway. However, the sensitivity and specificity of nuclear β-catenin expression are limited. Its transcriptional product, glutamine synthetase (GS) expression, is a reliable biomarker of the β-catenin mutation, while GS expression in human HCC is not always associated with β-catenin mutation (24–26). Therefore, the diagnosis of β-catenin mutation should be confirmed by the expression of β-catenin and GS by immunohistochemical analysis.

No stable serological or genomic biomarkers of the β-catenin mutation have been found to date due to the high heterogeneity of HCC. Moreover, the diagnosis of HCC depends not only on postoperative histologic examination, but also on imaging methods (3, 27). Thus, an accurate and noninvasive β-catenin mutation before surgery plays an important role in the prognostic assessment and selection of patients for anti-PD-1 therapy.

Signal intensity in liver-specific contrast-enhanced magnetic resonance imaging (MRI) is correlated with genetic alterations and molecular expression of HCC (28–32). Kitao et al. (30) suggested that the β-catenin mutation in HCC might show distinctive imaging findings in hepatobiliary phase (HBP) images. Nevertheless, these immunohistochemical predictors are difficult to detect using preoperative imaging methods. Radiomics is a rapidly growing methodology that permits digital decoding of medical images into multidimensional radiological features for noninvasive profiling of tumors. Several studies on HCC have demonstrated that unviewable radiomic features are closely associated with histopathologic features, especially in hepatobiliary-specific contrast-enhanced MRI. Feng et al. (33) constructed a radiomic feature-based nomogram using gadoxetic acid-enhanced MRI to achieve satisfactory preoperative prediction of microvascular invasion in patients with HCC. Wang et al. (34) showed that preoperative arterial and HBP imaging radiomic features could be a reliable biomarker to evaluate the CK19 status of HCC.

To our knowledge, no study has investigated the radiomics model of β-catenin mutation prediction. In this study, we aimed to determine the performance of a radiomic feature-based model using gadobenate dimeglumine (Gd-BOPTA)-enhanced MRI to predict β-catenin mutation and assist in the selection of optimal therapeutic strategies for patients with HCC.

## Materials and methods

### Ethics statements

Our study was approved by the Ethics Review Board of Eastern Hepatobiliary Surgery Hospital. The requirement for informed consent was waived because no protected health information was needed.

### Study design and patient cohort

**Figure 1:**
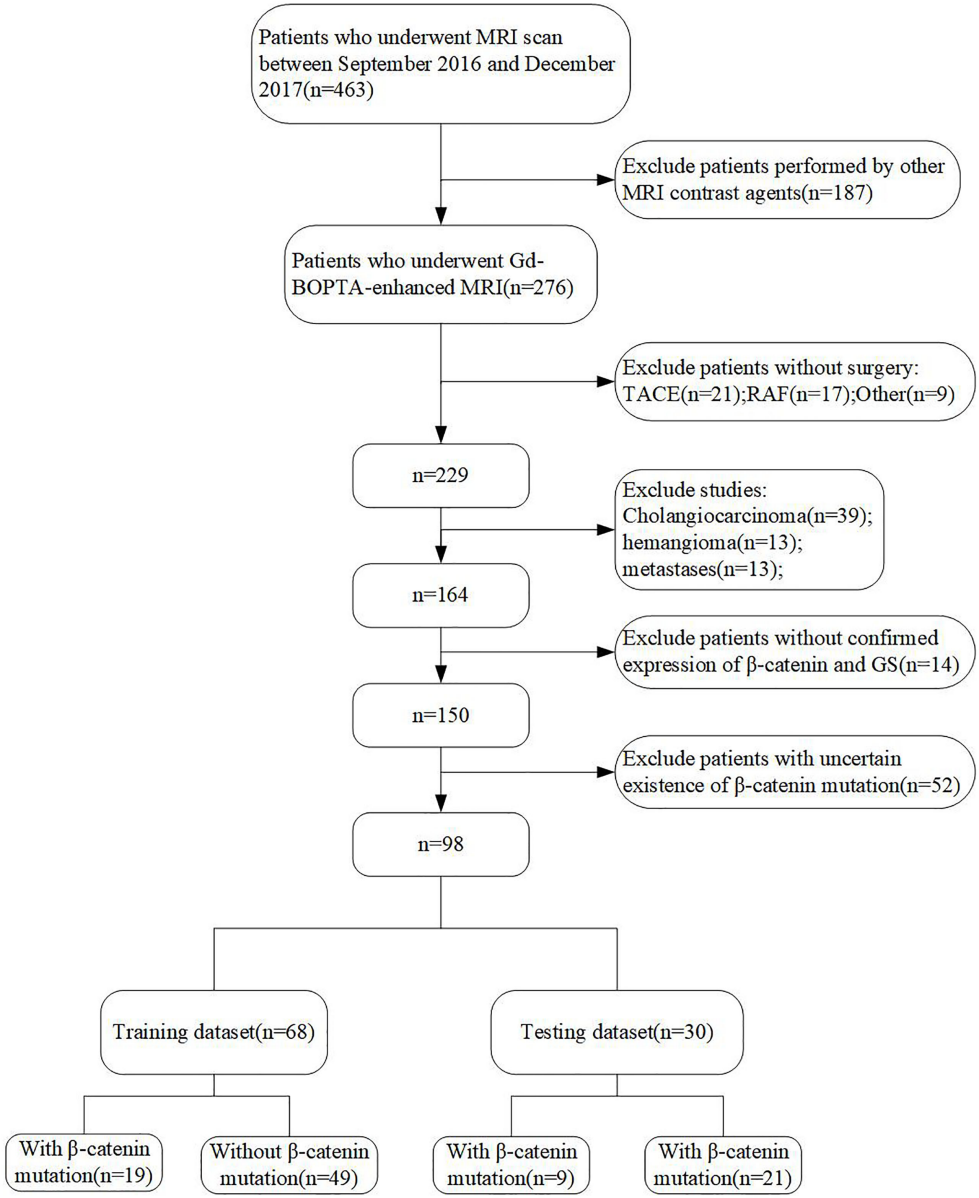
Flow chart of the study population. HCC, hepatocellular carcinoma; RAF, radiofrequency ablation; TACE, transcatheter arterial chemoembolization.

Between September 2016 and June 2017, 463 patients who underwent preoperative MRI at the Eastern Hepatobiliary Surgery Hospital were retrospectively enrolled and analyzed. Inclusion and exclusion criteria are shown in **Figure 1**.

### Magnetic resonance imaging

Gd-BOPTA-enhanced MRI was performed using GE Optima MR360 1.5 T. After injecting Gd-BOPTA (0.1 mmoL/kg; MultiHance, Braccom) into patients’ cubital veins at a flow rate of 2.0 mL/s, enhanced scanning of the arterial phase (AP, 22–25 seconds), portal venous phases (PVP, 50–60 seconds), and delayed phases (DP, 90–120 seconds) was performed, respectively. The scanning of HBP imaging was completed 60 minutes after injecting patients with normal liver function and 120 minutes after injecting patients with impaired function. The diagnosis of impaired liver function was made by clinical physicians. The scanning parameters are presented in **Table 1**.

**Table 1:** Sequences and parameters of Gd-BOPTA dynamic-enhanced MRI.

Sequences	TR/TE (msec)	FOV (mm)	Thickness (mm)	Flip angle	Matrix
T1WI	190/4.3 (2)	420×420	6	80	256×160
T2WI	6667/85	420×420	6	160	320×224
DCE	3.7/1.7	420×420	2.5	15	256×192
HBP	3.7/1.7	420×420	2.5	15	256×192

### Pathological examination

All tumor sections were reviewed by two experienced pathologists with 5–10 years of experience in HCC. Each tumor sample was first sectioned and then stained with hematoxylin-eosin. To determine the β-catenin mutation, the expressions of β-catenin and GS were examined by immunohistochemical analysis. In our study, the positive expressions of β-catenin and GS were attributed to the activation of β-catenin. However, no expression of β-catenin or GS was considered as HCC without a β-catenin mutation. HCC with a β-catenin mutation was shown in **Supplementary Figure 1** and without a β-catenin mutation was shown in **Supplementary Figure 2**.

### Magnetic resonance imaging signatures and clinical factor acquisition

Clinical factors (CFs), which included sex, age, alpha-fetoprotein, hepatitis B surface antigen (HBsAg), hepatitis B e antigen, and cirrhosis, were collected from patients’ electronic medical records. Based on the Liver Imaging Reporting and Data System (2017) (LI-RADS-2017), two abdominal radiologists with >10 years of experience assessed the radiological features and worked in consensus. MRI signatures included the tumor size, tumor morphology, capsule, margins, rim, and peritumoral enhancement in the AP, hypointensity, and peritumoral hypointensity in the HBP.

### Region of interest segmentation and feature extraction

**Figure 2:**
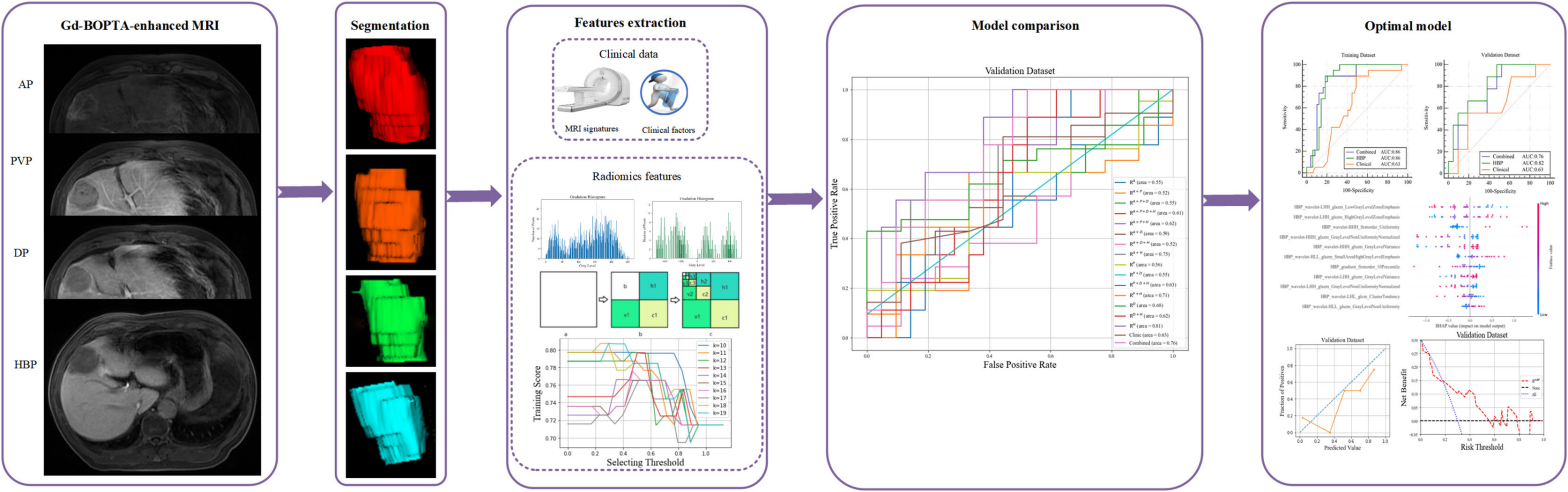
The workflow of the study.

Region of interest (ROI) segmentation was performed by a radiologist with 5 years of work experience and validated by a radiologist with >10 years of work experience using ITK-SNAP software (www.itk-snap.org). ROIs in AP, PVP, DP, and HBP images were delineated on each slice of the lesions and three-dimensional ROIs were generated accordingly (**Figure 2**). Overall, 1674 radiomic features were extracted from each MRI phase, which included 324 first-order features, 432 gray-level co-occurrence matrix features, 288 gray-level size zone matrix (GLSZM) features, 288 gray-level run length matrix features, 90 neighboring gray-tone difference matrix features, and 252 gray-level dependence matrix features. All radiomic features were extracted using the open-source software Pyradiomics (https://pyradiomics.readthedocs.io/en/latest/index.html). Features in each of the MRI sequences were combined and categorized into different groups.

### Feature selection and model construction

Patients were randomly allocated into a training/validation set (n=68) and a validation set (n=30) at a ratio of 7:3. The corresponding features were combined and categorized into four groups:

1. group 1 (n=4): features from one phase, named R^AP^, R^PVP^, R^DP^, and R^HBP^ (1674 features for each type);
2. group 2 (n=6): features from any two phases, named R^AP+PVP^, R^AP+DP^, R^AP+HBP^, R^PVP+DP^, R^PVP+HBP^, and R^DP+HBP^ (3348 features for each type);
3. group 3 (n=4): features from any three phases, named R^AP+PVP+DP^, R^AP+PVP+HBP^, and R^PVP+DP+HBP^,and R^AP+DP+HBP^ (5022 features for each type); and
4. group 4 (n=1): features from any four phases, named R^AP+PVP+DP+HBP^ (6696 features for this type).

All the features in different groups were first analyzed using the F-test. The top 30 features in each phase and ROI were selected based on the results of the F-test. For the clinical model, all 14 CF and MRI signatures were analyzed by the F-test, and the top five features were chosen. Next, features in 15 types of combinations and clinical models were selected by linear support vector classifier (LSVC), and were then cross-validated by a 10-fold cross-validation using the training set to obtain the optimal parameter.

We chose the LSVC as the only classifier in all radiomic and clinical models. The predictive power of these models was evaluated by the area under the receiver operating characteristic curve (AUC), accuracy, sensitivity, and specificity. The best radiomics model for predicting β-catenin mutation was determined using the validation dataset by comparing the AUC values. A combined model that integrated the radiomic signature and clinical characteristics was also developed using the LSVC. Calibration curves were applied to analyze the performance of the best model, and a decision curve analysis was used to determine the clinical usefulness. Additionally, a local interpretability technique called Shapley additive explanation (SHAP) was used to break down predictions and show the impact of each feature. This assessment was based on SHAP values (Shap0.32.1), which were equal to the prediction from the original forecasted value minus the deletion of the feature. Positive results signified that the feature supported the prediction, whereas negative values signified that the prediction was not supported.

### Statistical analysis

For baseline characteristics, continuous variables were analyzed using the t-test, and categorical variables were analyzed using the chi-square test or Fisher exact test. The AUC was compared using the DeLong test. Statistical analysis was performed using SPSS (version 22.0; IBM Corp.) and MedCalc (version 19.4.1). Statistical significance was set at *p*<0.05.

## Results

### Patient characteristics

The final cohort included 98 patients, who were randomly divided into training and validation datasets at a ratio of 7:3 (**Figure 1**). The longest interval between the MRI examination and surgery was approximately 2 weeks. The clinical characteristics and MRI features of all patients are shown in **Table 2**. There were no significant differences between the training and validation cohorts. In both datasets, most of the patients with HCC were male (85.3% and 83.3%, respectively), and the median ages were 55.63 and 54.13 years, respectively. The positive incidences of a β-catenin mutation were similar in the training and validation datasets (27.9% and 30%, respectively).

**Table 2:** Baseline clinical characteristics of the training and validation cohort.

Characteristic	Training dataset (N=68)	Validation dataset (N=30)	*P* value
Sex, N (%)			0.770^*^
Female	10 (14.7%)	5 (16.7%)	
Male	58 (85.3%)	25 (83.3%)	
Age, median ± SD (years)	55.63 ± 10.77	54.13 ± 11.90	0.540
Maximum diameter of tumor, median ± SD (mm)	41.60 ± 20.26	42.27 ± 18.76	0.879
Alpha fetoprotein, N (%)			0.816^*^
<20ng/ml	34 (50%)	17 (56.7%)	
20ng/ml-400ng/ml	27 (39.7%)	10 (33.3%)	
>400ng/ml	7 (10.3%)	3 (10%)	
HBsAg, N (%)			0.770^*^
Negative	10 (14.7%)	5 (16.7%)	
Positive	58 (85.3%)	25 (83.3%)	
HBeAg, N (%)			0.590
Negative	49 (72.1%)	20 (66.7%)	
Positive	19 (27.9%)	10 (33.3%)	
Cirrhosis, N (%)			0.743
Negative	18 (26.5%)	7 (23.3%)	
Positive	50 (73.5%)	23 (76.7%)	
Capsule, N (%)			0.506
Negative	54 (79.4%)	22 (73.3%)	
Positive	14 (20.6%)	8 (26.7%)	
Arterial rim enhancement, N (%)			0.219
Negative	43 (63.2%)	15 (50%)	
Positive	25 (36.8%)	15 (50%)	
Arterial peritumoral enhancement, N (%)			0.697^*^
Negative	63 (92.6%)	27 (90%)	
Positive	5 (7.4%)	3 (10%)	
Tumor margin, N (%)			0.810
Smooth	21 (30.9%)	10 (33.3%)	
Nonsmooth	47 (69.1%)	20 (66.7%)	
Tumor hypointensity on HBP, N (%)			\
Yes	68 (100%)	30 (100%)	
No	0	0	
Peritumoral hypointensity on HBP, N (%)			1.00^*^
Absent	59 (86.8%)	26 (86.7%)	
Present	9 (13.2%)	4 (13.3%)	
Shape, N (%)			0.095^*^
Round	15 (22.1%)	14 (46.7%)	
Oval	11 (16.2%)	4 (13.3%)	
Lobular	32 (47.1%)	8 (26.7%)	
Irregular	10 (14.7%)	4 (13.3%)	
β-catenin mutation, N (%)			0.835
Absent	49 (72.1%)	21 (70%)	
Present	19 (27.9%)	9 (30%)	

^*^ Calculated by Fisher’s exact test.

### Performance of the clinical model

In total, 14 clinical characteristics and MRI features were used to construct the clinical model. After feature selection, four features including sex, HBsAg, age, and peritumoral hypointensity in the HBP were included in the clinical model. The AUCs of the clinical model were 0.63 (95% confidence interval [CI]: 0.50–0.74) in the training dataset and 0.63 (95% CI: 0.54–0.80) in the validation dataset.

### Radiomics signature calculation

We performed LSVC modeling on AP, PVP, DP, and HBP features to explore the value of β-catenin mutation discrimination. For group 1, 12, four, four, and 11 features were selected for R^AP^, R^PVP^, R^DP^, and R^HBP^ model construction, respectively. For the biphasic and triphasic MRI images, the number of features selected as putatively effective features ranged from 2 to 16. Twelve features were selected for group 4. The details are shown in **Table 3**.

**Table 3:** Selected radiomics features of the proposed models.

Different model	Firstorder	GLCM	GLDM	GLRLM	GLSZM	NGTDM	Total
R^AP^	1	7	0	2	2	0	12
R^PVP^	0	0	0	1	3	0	4
R^DP^	3	0	0	0	1	0	4
R^HBP^	2	1	0	0	8	0	11
R^AP+PVP^	2	2	1	1	6	0	12
R^AP+DP^	2	0	0	1	2	0	5
R^AP+HBP^	1	2	0	2	10	0	15
R^PVP+DP^	2	0	0	3	4	0	9
R^PVP+HBP^	1	1	0	0	10	0	12
R^DP+HBP^	1	1	0	1	5	0	8
R^AP+PVP+DP^	1	0	0	2	4	0	7
R^AP+PVP+HBP^	2	2	0	3	9	0	16
R^AP+DP+HBP^	1	0	0	0	1	0	2
R^PVP+DP+HBP^	0	0	0	4	5	0	9
R^AP+PVP+DP+HBP^	1	0	0	4	6	0	11

### Performance of the proposed models

**Figure 3:**
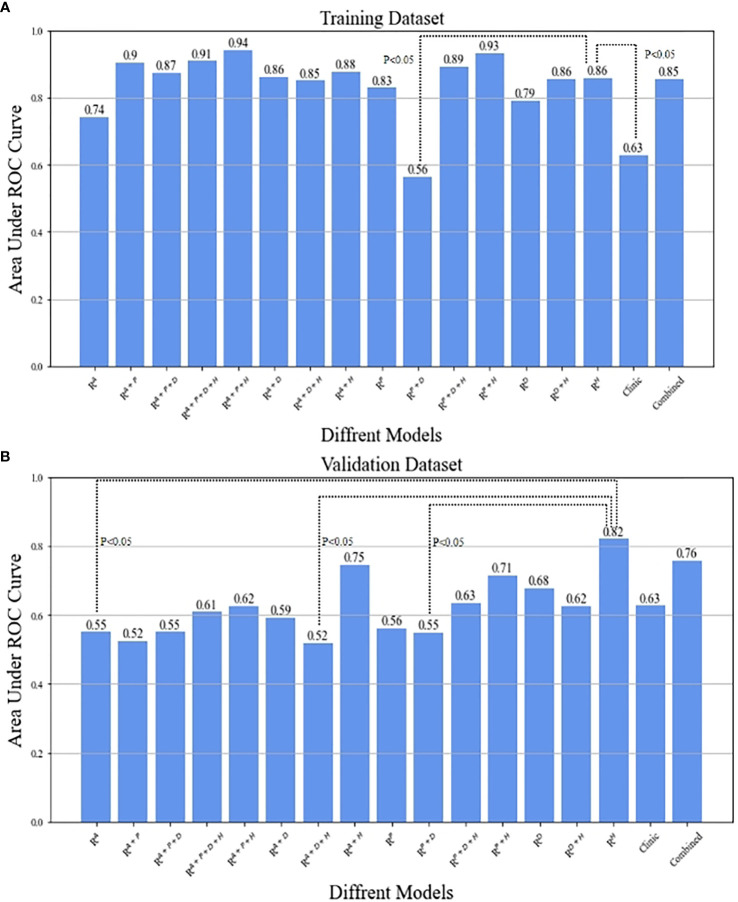
The comparison between R^HBP^ radiomics model in predicting β-catenin mutation and the other models by means of Delong test in the training **(A)** and validation **(B)** datasets.

All 15 types of radiomics models were compared to determine the best phases or combinations. The AUCs for all combinations of radiomics models are displayed in **Table 4**. The model with the best performance of was seen in the R^HBP^ radiomics model in the validation datasets, with AUCs of 0.82 among the R^AP^ (AUC=0.55), R^PVP^ (AUC=0.56), and R^DP^ radiomics models (AUC=0.68). For the biphasic MRI image, R^AP+HBP^, R^PVP+HBP^, and R^AP+PVP^ achieved excellent performance in the training datasets, but only the R^AP+HBP^ and R^PVP+HBP^ radiomics models retained a moderate AUC (AUC=0.75 and 0.71, respectively) in the validation datasets. Additionally, the radiomics models did not perform better in the tri-phasic or quad-phasic MRI images in the validation datasets (**Figure 3**).

**Table 4:** Predictive performance of the proposed models.

Different model	Training dataset				Validation dataset			
	AUC (95%CI)	ACC	SEN	SPE	AUC(95%CI)	ACC	SEN	SPE
R^AP^	0.74 (0.62-0.84)	0.69	0.68	0.69	0.55 (0.35-0.73)	0.70	0.44	0.81
R^PVP^	0.82 (0.71-0.91)	0.71	0.95	0.61	0.56 (0.36-0.74)	0.46	0.33	0.52
R^DP^	0.79 (0.67-0.88)	0.69	0.79	0.65	0.68 (0.48-0.83)	0.36	0.44	0.33
R^HBP^	0.86 (0.75-0.93)	0.75	1	0.65	0.82 (0.63-0.93)	0.73	0.67	0.76
R^AP+PVP^	0.90 (0.80-0.96)	0.80	0.89	0.76	0.52 (0.48-0.73)	0.60	0.33	0.71
R^AP+DP^	0.86 (0.75-0.93)	0.69	0.95	0.59	0.59 (0.60-0.82)	0.40	0.66	0.28
R^AP+HBP^	0.87 (0.77-0.94)	0.76	0.89	0.71	0.75 (0.61-0.86)	0.63	0.67	0.62
R^PVP+DP^	0.47 (0.43-0.68)	0.75	0.84	0.71	0.55 (0.40-0.65)	0.60	0.33	0.71
R^PVP+HBP^	0.93 (0.84-0.97)	0.81	0.95	0.76	0.71 (0.52-0.84)	0.60	0.56	0.61
R^DP+HBP^	0.85 (0.75-0.92)	0.69	0.84	0.63	0.62 (0.56-0.79)	0.57	0.67	0.52
R^AP+PVP+DP^	0.87 (0.77-0.94)	0.82	0.89	0.79	0.55 (0.52-0.76)	0.70	0.44	0.81
R^AP+PVP+HBP^	0.94 (0.85-0.98)	0.81	0.89	0.78	0.62 (0.54-0.78)	0.60	0.44	0.67
R^AP+DP+HBP^	0.85 (0.74-0.92)	0.76	0.89	0.71	0.52 (0.47-0.72)	0.50	0.67	0.43
R^PVP+DP+HBP^	0.89 (0.79-0.95)	0.83	0.74	0.88	0.63 (0.57-0.81)	0.66	0.44	0.76
R^AP+PVP+DP+HBP^	0.90 (0.81-0.96)	0.85	0.79	0.88	0.60 (0.51-0.82)	0.63	0.33	0.76
CF	0.63 (0.50-0.74)	0.57	0.89	0.45	0.63 (0.54-0.80)	0.3	0.44	0.24
Combined	0.86 (0.75-0.93)	0.69	0.89	0.61	0.76 (0.57-0.89)	0.63	0.56	0.67

CF, clinical factor; AP, arterial phase; PVP, portal venous phase; DP, delay phase; HBP, hepatobiliary phase; AUC, area under the curve; 95%CI, 95% confidence index; ACC, accuracy; SEN, sensitivity; SPE, specificity.

**Figure 4:**
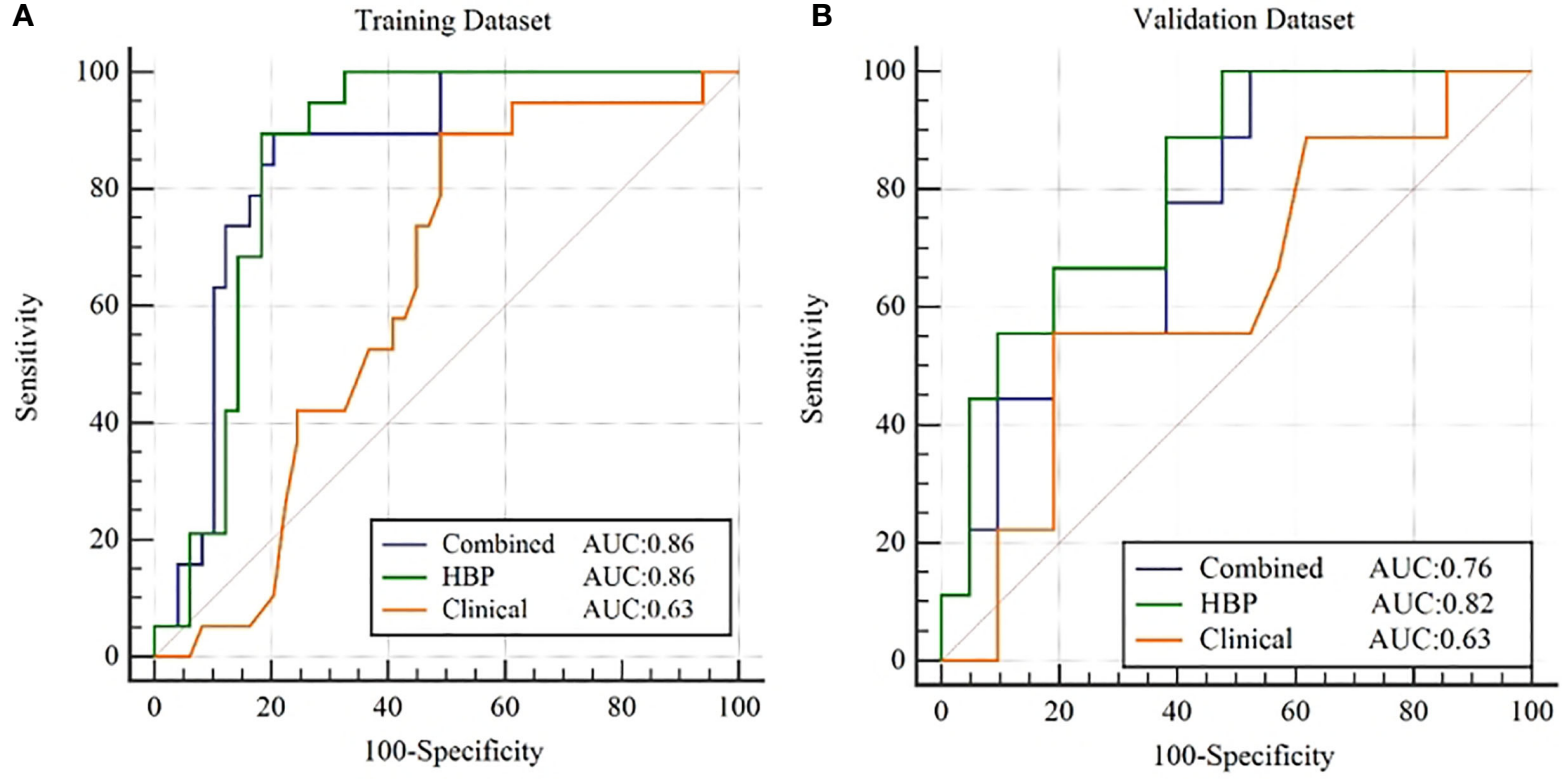
ROC curves for β-catenin mutation prediction of the clinical model, R^HBP^ radiomics model and combined model in the training **(A)** and validation **(B)** dataset.

After combining clinical characteristics in the clinical model with radiomics features in the R^HBP^ radiomics model, the combined model did not perform better than the single HBP radiomics model in the differentiation of the β-catenin mutation, with AUCs of 0.86 in the training dataset and 0.76 in the validation dataset. Comparisons were made between the best-performing R^HBP^ radiomics model and the clinical model. The Delong test resulted in a *p*-value of 0.0043 in the training dataset (AUC: 0.86 versus [vs.] 0.63). Although there was no significant difference in the validation dataset (*p*=0.151), there was a trend in that the single HBP radiomics model showed better performance than the clinical model with a higher AUC in the validation dataset (**Table 4** and **Figure 4**).

**Figure 5:**
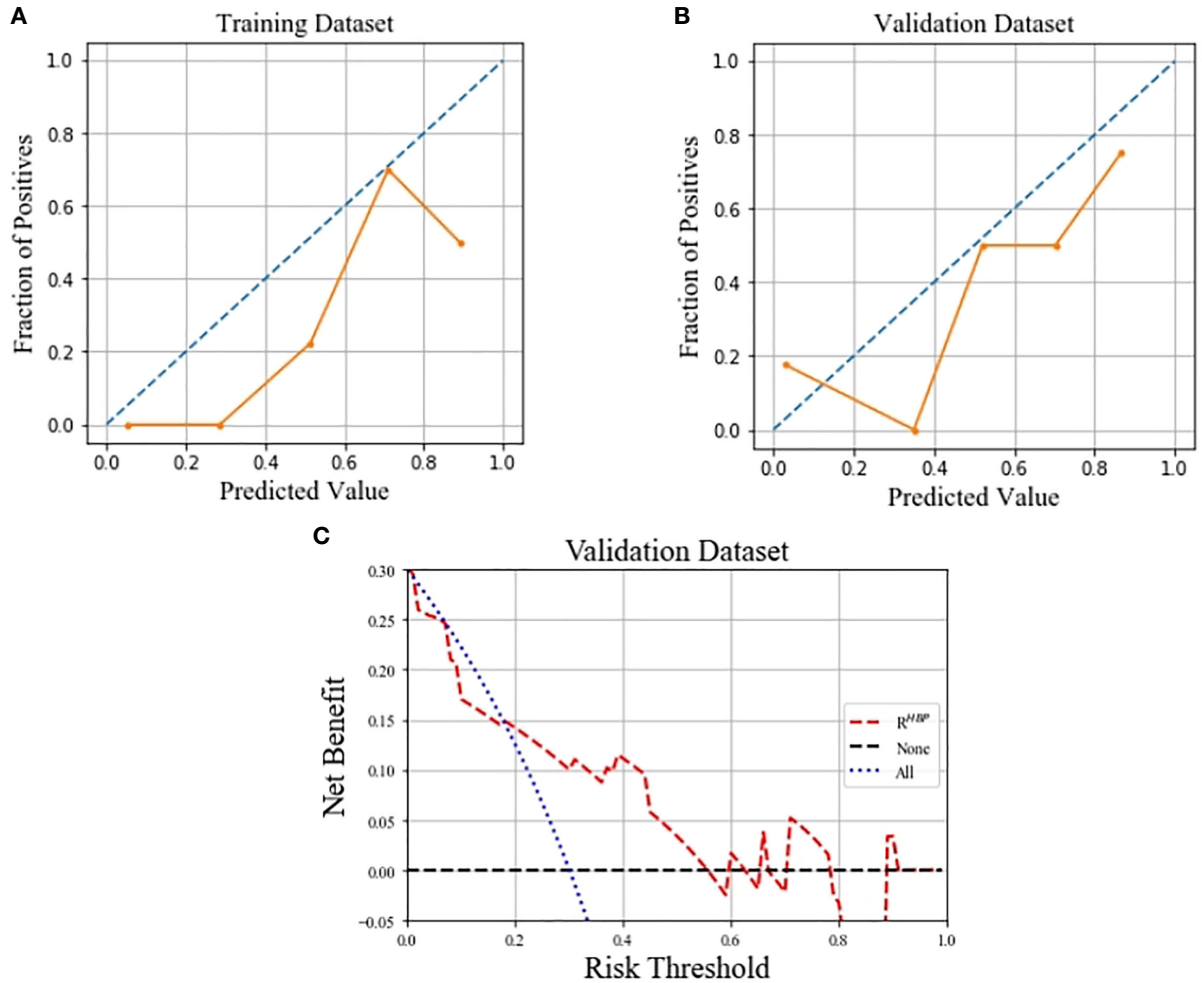
Calibration curves of the R^HBP^ radiomics model in predicting β-catenin mutation on the training **(A)** and validation **(B)** dataset, which demonstrated good agreement with the ideal curve. Decision curve analysis for the R^HBP^ radiomics model in the validation dataset **(C)**.

Calibration curves for the R^HBP^ radiomics model showed no significant difference between the predicted probabilities of the model and the ideal β-catenin mutation estimates in the training and validation datasets (*p*=0.081 and 0.454, respectively). The decision curve for the R^HBP^ radiomics model is shown in **Figure 5**. In our study, the net benefit could achieve 0.15, and the corresponding threshold probability of the curve was 0.18.

### Importance of the features in the R^HBP^ radiomics model

**Figure 6:**
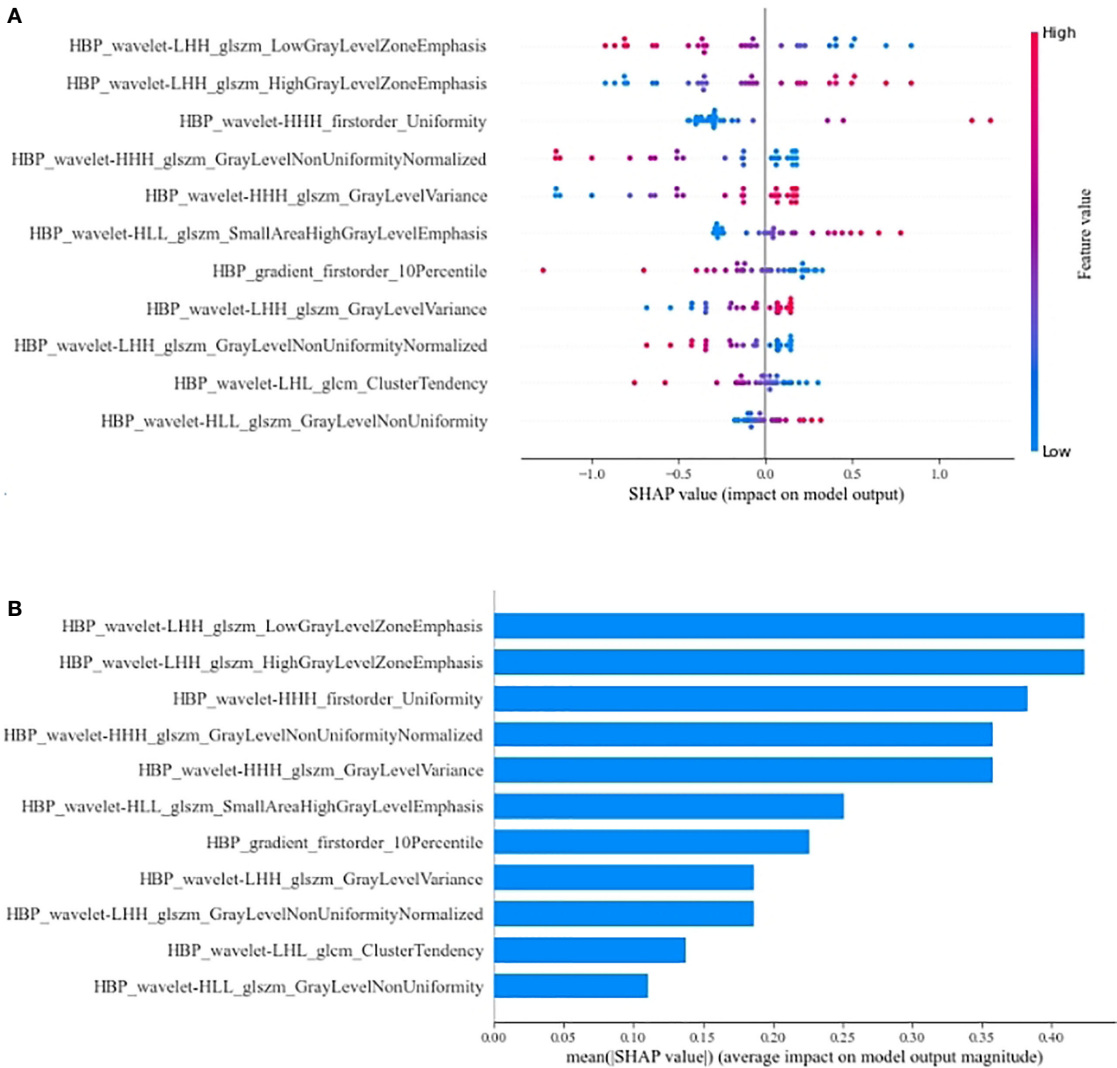
Summary plot **(A)** and bar plot **(B)** for the SHAP value of radiomics features on R^HBP^ radiomics model. LGLZE, low gray level zone emphasis; HGLZE, high gray level zone emphasis.

We further explored the interpretability of the R^HBP^ radiomics model. The SHAP values for the features were obtained to explore their contributions to the model (**Figure 6**). The most important radiomics features were LGLZE and HGLZE based on GLSZM with the same SHAP values of 0.424. However, the difference between LGLZE and HGLZE was that the former supported the absence of the β-catenin mutation, whereas the latter supported the presence of the β-catenin mutation.

## Discussion

To our knowledge, this is the first study to develop and validate Gd-BOPTA-enhanced MRI radiomics models for predicting the β-catenin mutation in HCC. Among the single-phase radiomics models in our study, the R^HBP^ radiomics model outperformed the other models in both the training and validation datasets. Some models, including bi-phase radiomics or others, could achieve better performance in the training dataset, but gain reverse results in the validation dataset. Furthermore, we established a combined model including radiomics from the best R^HBP^ radiomics model and features from a clinical model. However, the results showed that the combined model had the same performance as the single HBP radiomics model in the training datasets, but much worse performance in the validation datasets. This implies that radiomics based only on R^HBP^ images may aid in determining the activation of the β-catenin pathway effectively. The Hosmer-Lemeshow test of the training and validation datasets showed that the predicted curve was aligned with the ideal curve. Decision curve analysis in our study showed that the therapy strategy based on the R^HBP^ radiomics model was clinically useful.

We developed a clinical model combining clinical baseline factors and Gd-BOPTA-enhanced MRI features that demonstrated poor performance for preoperative prediction of the β-catenin mutation. Thus, it is difficult to distinguish the status of β-catenin by the macroscopical MRI features. Some scholars (30, 31, 35) have analyzed imaging findings, including a high enhancement ratio on R^HBP^ images, and a high apparent diffusion coefficient on diffusion-weighted imaging may be useful for identifying HCCs with the β-catenin mutation. While, these semi-quantitative parameter features cannot accurately assess the properties of the whole tumor. Radiomics can provide quantitative analysis of tumors. In our study, all the patients enrolled were showed hypointensity on R^HBP^ images, including patients with β-catenin mutation. However, the R^HBP^ radiomics model has achieved the best performance among all 15 types of radiomics models. We believe that more information related to the existence of the β- catenin mutation has been contained in the R^HBP^ images. Moreover, compared with visual and subjective imaging characteristics, radiomics can deeply excavate the information and provide a better prediction of the β-catenin mutation.

Many previous studies (31, 32) have demonstrated an intense correlation between signal intensity on the HBP images after liver-specific MR contrast administration and the expression of membranous uptake transporter organic anion-transporting polypeptide (OATP) 1B3. Additionally, the expression of OATP1B3 is due to the activation of β-catenin and/or hepatocyte nuclear factor 4α (36). Kitao et al. (30) reported a positive correlation between the expression of β-catenin and OATP1B3. Their results also showed that HCCs with the β-catenin mutation showed significantly higher enhancement ratios on HBP images than HCCs without. Reizine et al.’s study (35) of hepatocellular adenoma reported that liver-specific contrast uptake was strongly associated with activation of the β-catenin pathway. These findings were consistent with our results. Our results showed that the R^HBP^ radiomics model can achieve better performance than other single-phase and combined-phase radiomics models in the test datasets. By assigning a corresponding SHAP value to each feature in the best R^HBP^ radiomics model, we found that the most important features were LGLZE and HGLZE with the same SHAP value. LGLZE and HGLZE belong to GLSZM features, which describe the darkness and brightness of a lesion. LGLZE measures the distribution of lower gray-level zones, with a higher value indicating a greater proportion of lower gray-level values and size zones in the image, and HGLZE measures the distribution of the higher gray-level values, with a higher value indicating a greater proportion of higher gray-level values and size zones in the image. A larger value of LGLZE indicates a darker lesion on HBP images, which represents a low probability of HCC with the β-catenin mutation. Similarly, a larger value of HGLZE indicates a higher probability for the activation of the β-catenin pathway.

The current study has several advantages. First, unlike previous studies, we systematically evaluated and compared different MRI phases and their combinations. Additionally, we found an easy-to-use model that included only the proposed R^HBP^ radiomics signature to simplify prediction of the β-catenin mutation. Finally, the similar performance in the training and validation datasets made our R^HBP^ radiomics model more objective than others.

Our study also has limitations. First, since numerous potential participants did not undergo immunohistochemical analysis or preoperative liver-specific contrast-enhanced MRI, they were excluded, which may have created a selection bias and limited the validity of our results. Larger datasets should be used to validate the performance of our model in future studies. Second, the diagnosis of β-catenin activation was based on immunohistochemical analysis. The β-catenin mutation is supposed to be confirmed by polymerase chain reaction, but this was difficult to perform because of the retrospective nature of this study. However, previous studies (15, 16, 30) have shown that the expression of β-catenin and GS could represent the activation of β-catenin. We believe that the data in our study were sufficient to conclude that radiomics on HBP images was useful in preoperative individual prediction of HCCs with the β-catenin mutation. Our validation and training datasets were from the same center. Data from multiple centers should be used to assess the stability and generalizability of our findings.

In conclusion, the R^HBP^ radiomics model showed an excellent AUC with moderate sensitivity and specificity in both the training and validation cohorts. It can be used as an effective model to predicts HCCs with the β-catenin mutation preoperatively and thus may assist in the selection of appropriate decision-making for personalized medicine in patients with HCC.

## Methodology

- retrospective
- diagnostic or prognostic study
- performed at one institution

## Data availability statement

The raw data supporting the conclusions of this article will be made available by the authors, without undue reservation.

## Ethics statement

The studies involving human participants were reviewed and approved by the Ethics Review Board of Eastern Hepatobiliary Surgery Hospital. Written informed consent for participation was not required for this study in accordance with the national legislation and the institutional requirements.

## Author contributions

FZ, HD, and GQ designed the research. FZ, HD, and LZ performed the experiment. FZ, XL, LG, NJ, YJ, DH, and HZ collected and analyzed the data; FZ and HD prepared the original draft. LZ, GQ, and WC reviewed and edited the paper. All authors contributed to the article and approved the submitted version.

## Funding

This study was funded by the National Natural Science Foundation of China (82171929), National Key Research and Development Program of China [2019YFC0121903] and [2019YFC0117301], the Foundation of President of Nanfang Hospital [2021C007], Natural Science Funding of Guangdong Province [2018A0303130215], [2018A030313951] and [2019A1515011168].

## Acknowledgments

We would like to thank the whole study team for continuous support.

## Conflict of interest

The authors declare that the research was conducted in the absence of any commercial or financial relationships that could be construed as a potential conflict of interest.

## Publisher’s note

All claims expressed in this article are solely those of the authors and do not necessarily represent those of their affiliated organizations, or those of the publisher, the editors and the reviewers. Any product that may be evaluated in this article, or claim that may be made by its manufacturer, is not guaranteed or endorsed by the publisher.

## Supplementary material

Supplementary Figure 1 Photomicrographs show pathologic findings of HCCs with β-catenin mutation. **(A)** Hematoxylineosin staining (magnification, ×100) shows HCC with polygonal cells and round nucleus. At immunohistochemical analysis (magnification, ×200), tumor shows intense expression of, **(B)** nuclear β-catenin, **(C)** cytoplasmic GS.

Supplementary Figure 2 Photomicrographs show pathologic findings of HCCs without β-catenin mutation. **(A)** Hematoxylineosin staining (magnification, ×100) shows HCC with polygonal cells and round nucleus. At immunohistochemical analysis (magnification, ×200), tumor shows no definite expression of, **(B)** nuclear β-catenin, **(C)** cytoplasmic GS.
