# Supplementary figures and images for: Preoperative radiomics model using gadobenate dimeglumine-enhanced magnetic resonance imaging for predicting β-catenin mutation in patients with hepatocellular carcinoma: A retrospective study
Source: Front Oncol. 2022 Sep 16;12:916126. doi: 10.3389/fonc.2022.916126 (PMC9523364; doi:10.3389/fonc.2022.916126)

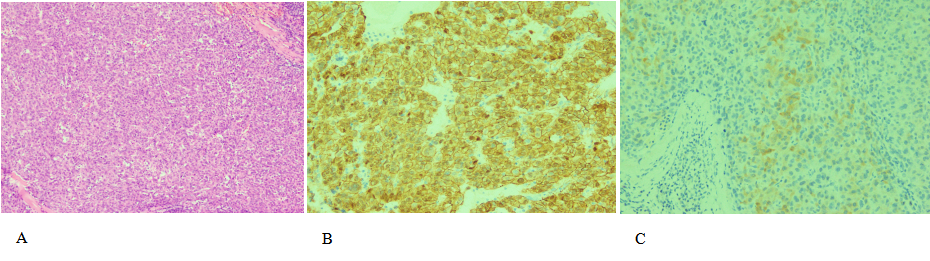

Supplement: Supplementary Figure 1 — Photomicrographs show pathologic findings of HCCs with β-catenin mutation. (A) Hematoxylineosin staining (magnification, ×100) shows HCC with polygonal cells and round nucleus. At immunohistochemical analysis (magnification, ×200), tumor shows intense expression of, (B) nuclear β-catenin, (C) cytoplasmic GS. [file Image_1.jpeg]

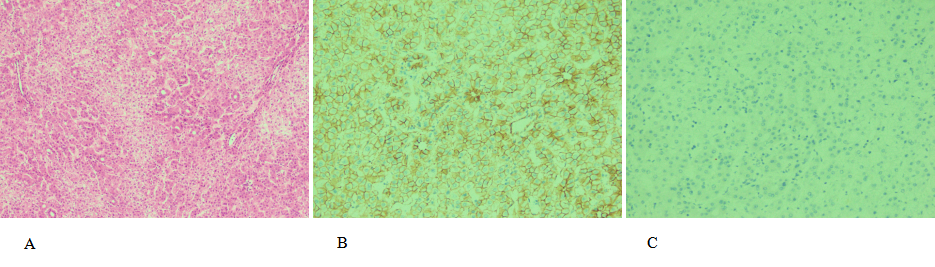

Supplement: Supplementary Figure 2 — Photomicrographs show pathologic findings of HCCs without β-catenin mutation. (A) Hematoxylineosin staining (magnification, ×100) shows HCC with polygonal cells and round nucleus. At immunohistochemical analysis (magnification, ×200), tumor shows no definite expression of, (B) nuclear β-catenin, (C) cytoplasmic GS. [file Image_2.jpeg]
